# Oxytocinergic neurons in the paraventricular nucleus mediate the analgesic effect of electroacupuncture in inflammatory pain

**DOI:** 10.1186/s13020-026-01513-x

**Published:** 2026-11-11

**Authors:** Jingyuan Liu, Xiaolei Wang, Qi Han, Yunyun Chen, Bofeng Zheng, Wei Yang, Bo Wang, Yantao Ma, Junlong Sun, Yongjun Zheng

**Affiliations:** 1 Department of Pain Management; Department of Rehabilitation Medicine; Shanghai Key Laboratory of Clinical Geriatric Medicine, Huadong Hospital, Fudan University, Shanghai, China; 2 Dapuqiao Community Health Service Center, Huangpu District, Shanghai, China

**Keywords:** Electroacupuncture, Oxytocinergic neurons, Paraventricular nucleus, Arcuate nucleus

## Abstract

**Background:**

Electroacupuncture (EA) is a well-established and effective analgesic intervention, particularly for inflammatory pain; however, the analgesic effect of EA is closely related to the stimulation frequency, amplitude, and acupoint. The underlying neural mechanisms remain incompletely understood. This study aimed to identify the central neural projections responsible for EA-induced analgesia and to investigate the mechanisms underlying its frequency-specific effects.

**Methods:**

In this study, we utilized a mouse model of carrageenan (CGN)-induced inflammatory pain to explore the analgesic effects of EA. In vivo fiber photometry and immunofluorescence were employed to monitor the activity of oxytocinergic neurons in the paraventricular nucleus (PVN). The role of neuronal activity in EA-induced analgesia was assessed through chemogenetic and pharmacological approaches.

**Results:**

We demonstrated that specific activation of PVN oxytocinergic neurons significantly alleviated pain behaviors in the inflammatory pain model mice. EA stimulation of the Stomach meridian 36 (ST36) activated PVN oxytocinergic neurons and exerted an antinociceptive effect in a frequency-dependent manner. Specifically, lower frequency EA stimulation induced more significant PVN oxytocinergic neuronal activity and analgesic effect, while higher frequency EA stimulation did not show significant neuronal activation or pain relief. Chemogenetic silencing of PVN oxytocinergic neurons completely abolished EA-induced analgesia. Additionally, we identified that oxytocinergic projections from the PVN to the arcuate nucleus (ARC) were activated only under lower frequency EA stimulation, while higher frequency EA stimulation failed to exert this effect. What’s more, locally blocking the target of oxytocin in the ARC significantly attenuated the analgesic effect of EA.

**Conclusions:**

Our findings highlight the critical role of PVN oxytocinergic neurons in mediating the frequency-specific analgesic effects of EA, which provide important evidence for parameter selection in clinical EA treatment for pain.

**Supplementary Information:**

The online version contains supplementary material available at https://doi.org/10.1186/s13020-026-01513-x.

## Introduction

Pain is a complex sensory and emotional experience that arises from actual or potential tissue damage. It is not only a symptom but also a disease encompassing multiple types and varying degrees of severity. Inadequate pain management not only has a great impact on the physical and psychological well-being of patients but also causes significant economic losses to society. In the clinical setting of pain treatment, pharmacotherapy is still the most common treatment at present [1]. However, studies have shown that pharmacological options are accompanied by side effects. Overdose of non-steroidal anti-inflammatory drugs (NSAIDs) is associated with several serious adverse effects, including hypertension, cardiovascular disease, kidney injury, and gastrointestinal complications [2]. Likewise, opioids produce dose-dependent side effects such as nausea, dizziness, and respiratory depression, and their long-term use can lead to tolerance and diminished analgesic efficacy [3]. These limitations highlight the urgent need for complementary or alternative non-pharmacological interventions for clinical pain management.

Acupuncture, a therapy with a history of more than 2,500 years in China, has been recognized as a potent and safe approach for pain relief [4]. Although it originated in China, acupuncture has gained increasing global acceptance, and its treatment for inflammatory pain has shown good results. According to the World Health Organization (WHO), acupuncture is recommended for the treatment of 77 diseases. And 16 of these diseases are related to inflammatory pain [5]. EA is a therapy based on acupuncture combined with pulsed electrical stimulation, which offers additional advantages such as reproducibility and controllable stimulation parameters. Previous studies have shown that the analgesic effect of EA is closely related to the stimulation parameters, especially the stimulation frequency [6]. Lower frequency EA stimulation, with its faster onset and longer duration of analgesia, has a better overall analgesic effect than higher frequency EA stimulation [7–9]. In the central mechanisms of EA-induced frequency-specific analgesia, the ARC of the hypothalamus plays an important role. Studies revealed that destruction of the ARC abolishes the effects of pain relief induced by low-frequency EA rather than high-frequency EA [10]. However, how EA at different frequencies affects the hypothalamic circuits and produces distinct analgesic effects remains poorly understood.

An accumulating body of evidence suggests that the hypothalamic neuropeptide oxytocin (OXT) exerts significant modulatory influences on pain processing [11]. In mammals, oxytocinergic neurons are predominantly located in the supraoptic nucleus (SON) and PVN of the hypothalamus, and their nerve fibers project to the posterior lobe of the pituitary gland, releasing OXT into the peripheral circulation to regulate diverse physiological functions [12]. There is a specific population of oxytocinergic neurons localized exclusively to the PVN that project widely to different areas of the brain and spinal cord [13, 14], which play a vital role in the central nervous system. A previous study found that delivery of OXT markedly reduces persistent inflammatory pain in rats, and oxytocinergic neurons in the PVN play a key role in reducing nociceptive responses [15]. The PVN, positioned adjacent to the third ventricle, also has been identified as a key hypothalamic hub integrating pain-related signals [16]. Although oxytocinergic neurons are known to participate in nociceptive modulation, their role in EA-induced analgesia remains largely unclear.

As the target for OXT, oxytocin receptor (OXTR) is a 7-transmembrane G protein-coupled receptor capable of binding to either Gαi or Gαq proteins, activating a set of signaling cascades [17]. OXTRs are widely localized in many brain nuclei associated with the oxytocinergic fibers, and the OXT–OXTR system plays a critical role in regulating pain behaviors [14, 18]. Neurons in the ARC express a high number of oxytocin receptors and receive direct inputs from the PVN [19, 20]. Here, we hypothesized that OXTR may be the critical substance linking the modulation of the ARC by lower-frequency EA and the activity of oxytocinergic neurons in the PVN. These findings may provide important insights into the mechanism of frequency-dependent analgesia by EA stimulation.

## Materials and methods

### Animals

Male C57BL/6J mice were purchased from Shanghai Slake Experimental Animal Co., Ltd (Shanghai, China). Transgenic *hM4Di* (R26-LSL-Gi-DREADD, JAX#026219) mice were obtained from The Jackson Laboratory. Mice aged 8 to 12 weeks and weighing approximately 25 g were used for all experiments. Animals were housed in a pathogen-free environment under controlled temperature of 22–24℃ and a 12-h light–dark cycle, with ad libitum access to water and food. All experimental procedures were approved by the Institutional Animal Care and Use Committee of Fudan University and were conducted in accordance with the guidelines for the care and use of laboratory animals by the National Institutes of Health.

### Establishment of a CGN-induced inflammatory pain mouse model

The inflammatory pain model was established by CGN as previously described [21, 22]. The mice were anesthetized with inhalation of isoflurane for 2–3 min in a plastic induction box. A subcutaneous injection of 20 μL of 2% (W/V) λ-carrageenan (Sigma, St. Louis, MO, USA) dissolved in normal saline was administered into the plantar surface of the left hind paw of mice using a 25-gauge needle. Mice receiving an equivalent volume of sterile saline served as vehicle controls.

### Electroacupuncture

During EA stimulation, the mice were anaesthetized with 0.5%-1% isoflurane, and body temperature was maintained using a heating pad. A pair of unipolar stainless-steel needles was inserted at the left hindlimb ST36 acupoint to a depth of 3–4 mm. Electrical stimulation was delivered using a stimulator (Model 3800, A-M Systems) and 8 isolators (Model 3820, A-M Systems). The stimulation parameters were set at intensities of 0, 0.1, 0.5, or 2 mA, and frequencies of 2, 10, or 100 Hz, with a pulse width of 50 μs. The acupoint ST36 is located below the lateral side of the knee joint [23, 24].

### Mechanical allodynia test

Mechanical allodynia was assessed using the Dixon up–down method with von Frey filaments, which is the gold standard for assessing mechanical thresholds in mice [25, 26]. Mice were habituated to the testing environment for three consecutive days and allowed to acclimate for 30 min in individual Plexiglas chambers placed on a wire mesh floor before testing. A series of von Frey hairs was applied perpendicularly to the central plantar surface of the left hind paw. The test began with the application of a 0.16 g von Frey filament, and then the force increased or decreased according to the response. A brisk paw withdrawal or lifting was considered a positive response. After the first change in response, five additional stimuli were applied. The 50% withdrawal threshold was calculated using the Dixon up–down paradigm.

### Thermal hyperalgesia test

Thermal hyperalgesia was evaluated using the Hargreaves’ Apparatus (Ugo Basile) [27, 28]. Mice were habituated individually in a plexiglass box for 20 min before testing. A radiant heat source with 30% irradiation intensity was focused on the plantar surface of the left hind paw, and paw withdrawal latency was automatically recorded when the animal lifted or licked the paw. At least five trials were performed for each testing condition, and trials were conducted with 5 min intervals. The average latency of five trials was calculated and recorded.

### Conditioned place aversion (CPA) assay

CPA experiments were performed using a standard two-compartment apparatus that allowed a mouse to travel between the two chambers freely [29, 30]. The chambers were visually distinguished by distinct wall patterns (stripes or grids). The CPA procedure consisted of three phases: preconditioning, conditioning, and testing.

During the preconditioning phase (10 min), mice were allowed to freely explore both chambers. Animals spending more than 480 s or less than 120 s of the total time in either chamber were eliminated from the analysis. Then the conditioning phase immediately began after the preconditioning. In the conditioning phase (10 min per chamber), one chamber was paired with mechanical stimulation (0.16 g von Frey filament), while the other was paired with no stimulation. Each mechanical stimulus lasted 3–5 s at 30 s intervals. The order of chamber pairings and stimulation was counterbalanced.

During the testing phase (10 min), mice were allowed to move freely without stimulation. Time spent in each chamber was recorded during both the preconditioning and testing phases. Decreased time spent in a chamber during the testing phase relative to the preconditioning indicated aversion to that chamber. The CPA score was computed by subtracting the amount of time mice stayed in the stimulus-paired chamber during the testing phase from the time in the preconditioning phase. A higher CPA score reflects stronger aversive behavior.

### Intracranial viral injections and optic fiber implantation

For chemogenetic manipulation of the PVN oxytocinergic neurons, viral cocktails containing rAAV-mOXT-CRE-EGFP-WPRE-bGH pA (Brain VTA) and rAAV2/9-Ef1a-DIO-Gq-mCherry-WPRE (Brain VTA) or rAAV-mOXT-CRE-EGFP-WPRE-bGH pA and rAAV2/9-Ef1a-DIO-Gi-mCherry-WPRE (Brain VTA) viral cocktails (1:1) were stereotaxically injected into the bilateral PVN (anteroposterior (AP) − 0.60 mm, mediolateral (ML) ± 0.25 mm, dorsoventral (DV) − 4.8 mm) of the WT mice (400 nL per site). In the *hM4Di* mice, rAAV-mOXT-CRE-EGFP-WPRE-bGH pA was injected into the bilateral PVN (200 nL per site). Chemogenetic activation or inhibition was achieved 3 weeks post-injection by intraperitoneal administration of Clozapine-N-oxide (CNO) (1 mg/kg, Brain VTA). After all the behavioral tests, mice were fixed to verify the location of the virus injection.

To monitor PVN oxytocinergic neuronal activity, a 1:1 viral cocktail of rAAV-mOXT-CRE-EGFP-WPRE-bGH pA and rAAV-EF1a-DIO-GCaMP6m-WPRE-hGH polyA (Brain VTA) was injected into the left PVN (400 nL total volume). Optical fibers (105-μm core/125-μm cladding, Thinker Tech) were implanted in the left PVN or ARC (AP − 1.41 mm, ML − 0.25 mm, DV − 5.75 mm) after AAV injection. Calcium signals were recorded using a fiber photometry system (Thinker Tech). Fluorescence signal excitation used a 470 nm laser, while a 405 nm laser served as a reference to isolate movement-corrected signals from the 470 nm channel, and the laser power was adjusted to 50 μW. After all the recording tests, mice were fixed to verify the location of the fiber and the accurate placement of the virus injection.

### Cannula implantation and intra-ARC infusion

Cannula implantation and local L-368,899 infusion were prepared according to a previously described method [31]. The L-368,899 (MedChemExpress) was dissolved in sterile normal saline. A stainless-steel guide cannula (RWD) was implanted above the ARC region. To prevent clogging of the guide cannula, stainless-steel dummies (RWD), which extended 0.2 mm beyond the guide cannula, were inserted. At least one week for recovery after the cannula implantation, the mice were proceeded with subsequent experimental procedures.

Thirty minutes prior to EA stimulation, mice were anesthetized with isoflurane, and saline or L-368,899 was delivered into the ARC region using an injection cannula (RWD) extending 0.5 mm beyond the tip of the guide cannula. The L-368,899 (1.25 mM in 185 nL) or an equal volume of saline was administered into the ARC region. After all the behavioral tests, mice were fixed to verify the location of the cannula and the drug injection.

### Immunofluorescence staining

Mice were euthanized by CO_2_ inhalation and perfused transcardially with phosphate-buffered saline (PBS) followed by 20 mL of 4% paraformaldehyde (PFA). The brains were dissected and post-fixed in 4% PFA at 4 °C overnight and dehydrated using 30% sucrose buffer, and then sectioned at 40 μm using a freezing microtome (RWD). The sections were incubated with primary antibodies overnight at 4 °C, followed by secondary antibodies for 1.5 h at room temperature.

The following primary antibodies were used: rabbit anti-OXT (Abcam), rat anti-c-Fos (SYSY), chicken anti-GFP (Invitrogen), and goat anti-tdTomato (Sicgen). The secondary antibodies were labeled with Alexa Fluor 488, Alexa Fluor 594, and Alexa Fluor 647, purchased from Jackson Immunology.

### Drug administration

L-368,899 is a selective, non-peptide OXTR antagonist, which is one of the most commonly used drugs in animal research for the selective blockade of neural OXTR [32]. The dose of L-368,899 was based on previous studies [33]. L-368,899 was dissolved in saline and administered intraperitoneally (5 mg/kg) 30 min before EA administration. The same volume of saline was injected intraperitoneally into the control mice.

### Measure of paw volume

The paw volume of mice was measured using a toe volume meter (Beijing Zhishuduobao Biotechnology Co., Ltd.) immediately before, and 6 h and 24 h after subplantar CGN/saline injection as an index of inflammation.

### Statistical analysis

All data are expressed as mean ± standard error of the mean (SEM). Statistical analyses were conducted using GraphPad Prism 9.5.1 software. The Shapiro–Wilk test was used to assess the normality, and the Brown-Forsythe test was used to assess homoscedasticity. For normally distributed data, unpaired *t*-tests were used to compare the two groups; one-way ANOVA followed by Dunnett’s multiple comparisons test and two-way ANOVA followed by Bonferroni’s post hoc test or Dunnett’s multiple comparisons test was used for multiple comparisons. For non-normally distributed data, the Mann–Whitney test was used for unpaired comparisons, and the Kruskal–Wallis test followed by Dunn’s multiple comparisons test for multiple comparisons. Statistical significance was defined as *p* < 0.05, with levels indicated as *p < 0.05, **p < 0.01, and ***p < 0.001. The calculated statistical power for all key comparisons is above 0.8, indicating that the sample sizes are sufficient to detect the reported effects.

## Results

### Chemogenetic activation of the PVN oxytocinergic neurons exerts the analgesic effect in the CGN-induced inflammatory pain

We first established a CGN-induced inflammatory pain mouse model via plantar injection of CGN. Mice injected with CGN developed robust mechanical allodynia and thermal hyperalgesia in the ipsilateral paw, as characterized by reduced mechanical withdrawal threshold to von Frey stimulation as well as shortened latency to radiant heat–evoked paw withdrawal, compared to mice injected with saline (Figure S1A, Figure S1C). Both mechanical and thermal hypersensitivity peaked at approximately 6 h post-injection, persisted for nearly 5 days, and gradually returned to baseline within 7 days (Figure S1B, Figure S1D). CGN injection also induced visible paw edema (Figure S1E-F).

**Fig. 1:**
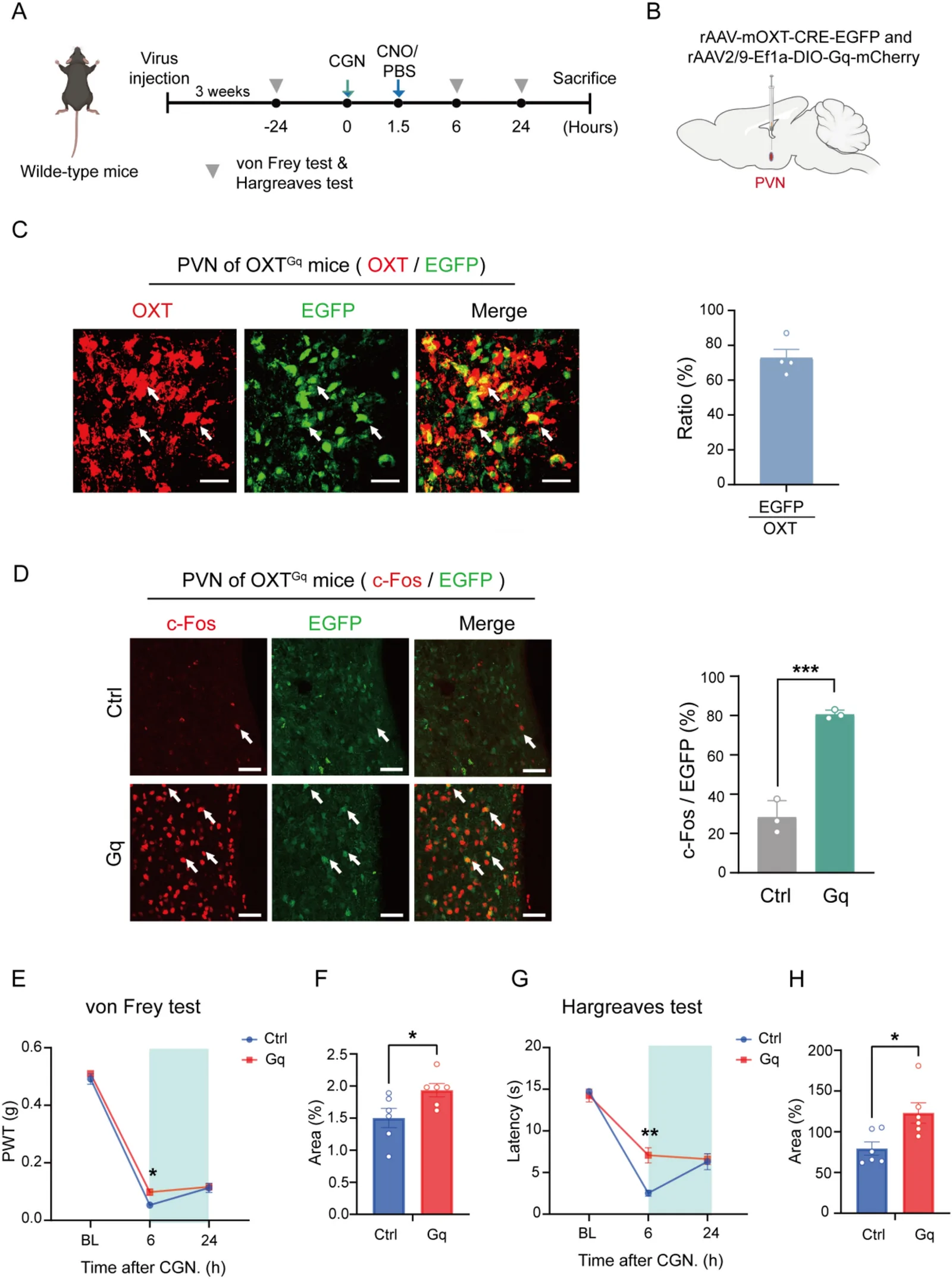
Chemogenetic activation of the PVN oxytocinergic neurons exerts the analgesic effect in the CGN-induced inflammatory pain. **A** Strategy to activate PVN oxytocinergic neurons in inflammatory pain mice. **B** Schematic of microinjection of rAAV-mOXT-CRE-EGFP and rAAV2/9-Ef1a-DIO-Gq-mCherry into the PVN region. **C** Representative images validate EGFP expression in immunofluorescent staining of OXT neurons in PVN of mice (scale bar = 25 μm). White arrows indicate co-localization. n = 4 mice. **D** Representative images validate c-Fos expression in the EGFP neurons of mice (scale bar = 50 μm). White arrows indicate EGFP neurons expressing c-Fos. n = 3 mice per group. **E** Mechanical pain threshold after CGN administration in the left paw of mice in the Gq or the Ctrl group, n = 6 mice per group. **F** Normalized AUC of PWT during 6 to 24 h from **E**, n = 6 mice per group. **G** The thermal pain threshold after CGN administration in the left paw of mice in the Gq or the Ctrl group, n = 6 mice per group. **H** Normalized AUC of latency during 6 to 24 h from **G**, n = 6 mice per group

Given accumulating evidence that the activity of oxytocinergic neurons in the PVN contribute to antinociceptive processing in inflammatory pain [13, 14, 33, 34], we sought to determine whether selective chemogenetic activation of oxytocinergic neurons in the PVN has an analgesic effect in the CGN-induced inflammatory pain model (Fig. 1A). We first assessed the neuronal activity changes after CGN injection. The PVN of mice was stained for OXT and c-Fos (a marker of neuronal activation [35]) after CGN or saline injection. The results demonstrated that CGN injection increased oxytocinergic neuron activity in the mouse PVN versus saline controls (Figure S1G-H). These findings suggest that under pain, oxytocinergic neurons become activated and may exert a self-protective role. To selectively activate oxytocinergic neurons in the PVN, a viral cocktail consisting of rAAV-mOXT-CRE-EGFP-WPRE-bGH and rAAV2/9-Ef1a-DIO-Gq-mCherry-WPRE (1:1) was bilaterally delivered into the PVN of mice (Fig. 1B). This approach restricted the expression of Cre recombinase and the excitatory DREADD receptor Gq to oxytocinergic neurons in the PVN of mice. Immunofluorescent staining performed 21 days after injection confirmed that EGFP-labeled neurons were largely colocalized with OXT-immunoreactive (OXT-ir) neurons (Fig. 1C), validating the cell-type specificity of our viral scheme. CNO was used to activate oxytocinergic neurons as the ligand of the Gq-coupled DREADD [36]. The expression of c-Fos was robustly increased in EGFP-positive neurons after intraperitoneal injection of CNO (1 mg/kg) in mice of the Gq group, contrasted with equal PBS administered to mice in the Ctrl group (Fig. 1D). These results verified that our chemogenetic paradigm effectively activated PVN oxytocinergic neurons in mice of the Gq group.

Activation of PVN oxytocinergic neurons following CGN injection, mice of the Gq group exhibited significantly elevated mechanical thresholds (Fig. 1E, F) and prolonged thermal withdrawal latencies (Fig. 1G, H) at 6 h compared with mice of the Ctrl group. However, the degree of CGN-induced paw swelling was similar between the two groups (Figure S2A-B). Together, these results demonstrate that chemogenetic excitation of oxytocinergic neurons in the PVN elicits analgesic effects in the CGN-induced inflammatory pain mouse model without influencing peripheral inflammation.

### Electroacupuncture promotes the activation of oxytocinergic neurons in the PVN

**Fig. 2:**
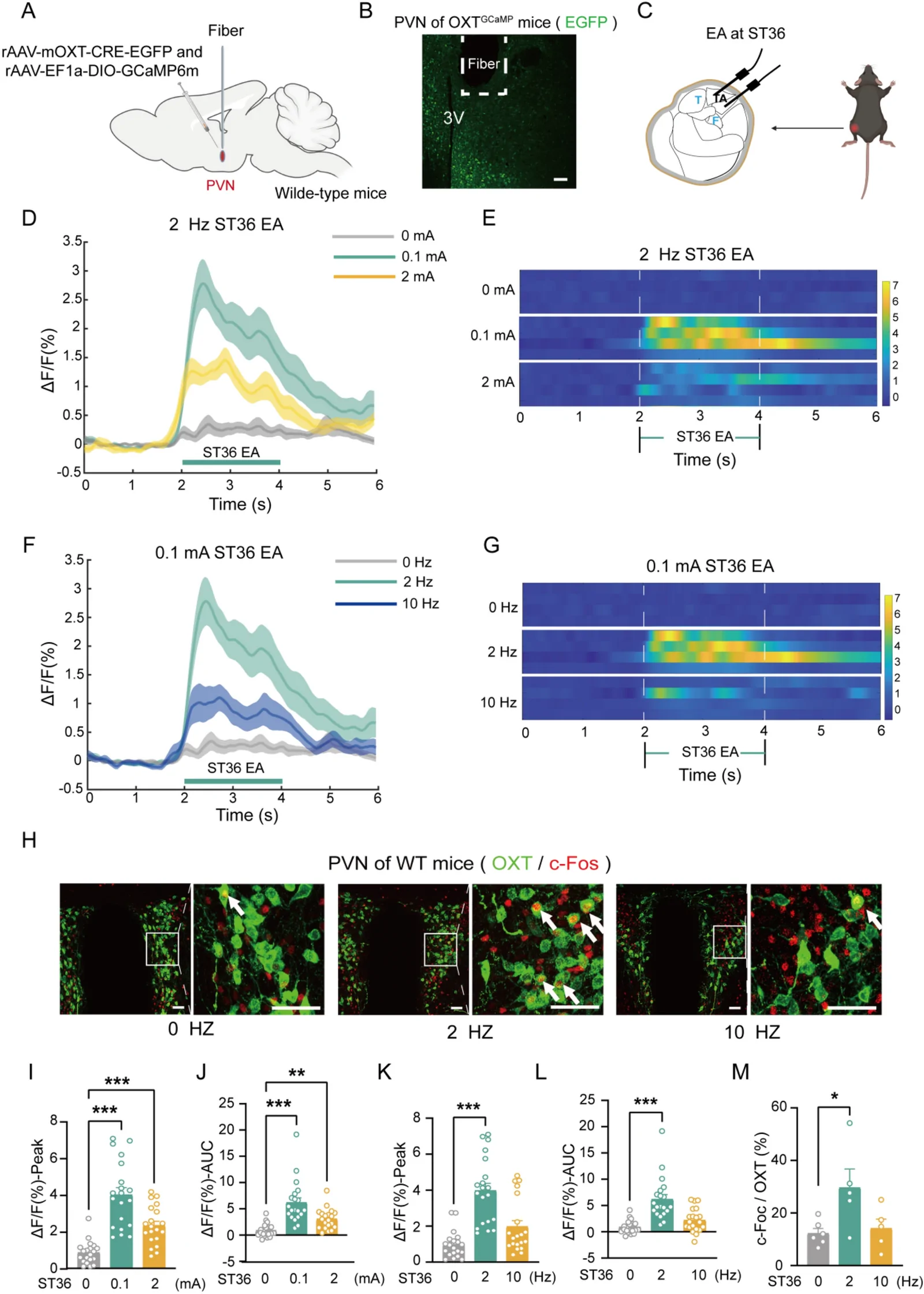
Electroacupuncture promotes the activation of oxytocinergic neurons in the PVN. **A** Schematic of microinjection of rAAV-mOXT-CRE-EGFP with rAAV-Ef1a-DIO-GCaMP6m and the optical fiber insertion into the PVN region. **B** Representative images validating viral expression and fiber input in PVN (scale bar = 100 μm). **C** Schematics showing EA at the ST36 acupoint. **D** Typical examples of traces and **E** heat maps of Ca^2+^ transients in PVN oxytocinergic neurons induced by EA stimulus of 2 Hz for 2 s. **F** Typical examples of traces and **G** heat maps of Ca^2+^ transients in PVN oxytocinergic neurons induced by EA stimulus of 0.1 mA for 2 s. **H** Representative immunofluorescent staining of OXT (green) and c-Fos (red) in the PVN of the mice after 0.1 mA EA stimulation with different frequencies (scale bar = 50 μm) and corresponding local enlarged views (scale bar = 50 μm). White arrows indicate oxytocinergic neurons expressing c-Fos. **I** Quantitative summary of Ca^2+^ transients in peak amplitude and **J** AUC before and after EA stimulation of 2 Hz with different intensities, n = 20 trials from 4 mice per group. **K** Quantitative summary of Ca^2+^ transients in peak amplitude and **L** AUC before and after EA stimulation of 0.1 mA with different frequencies, n = 20 trials from 4 mice per group. **M** Percentage of c-Fos-positive PVN OXT neurons after the EA stimulation, n = 5–6 mice per group

Given the above, activation of PVN oxytocinergic neurons exerted an analgesic effect; we went on to explore whether EA stimulation could activate oxytocinergic neurons in the PVN. We employed in vivo real-time Ca^2+^ photometry recording in the oxytocinergic neurons of the PVN region. The viral cocktails (1:1) of rAAV-mOXT-CRE-EGFP-WPRE-bGH and rAAV-EF1a-DIO-GCaMP6m-WPRE-hGH were stereotaxically injected into the left PVN of mice, and an optical fiber was implanted above the injection site to record real-time neuronal activity (Fig. 2A, B). The hindlimb ST36 acupoint has been chosen for EA stimulation (Fig. 2C), which was associated with pain treatment [37, 38]. Three weeks after viral injection, EA stimulus was applied at the ST36 acupoint under light isoflurane anesthesia, and the changes in GCaMP fluorescence in response to EA stimulation were recorded. The value of peak and area under the curve (AUC) were used to quantify the GCaMP signals, which respectively reflect the maximal and overall signal intensity.

Among the 2 Hz EA stimulation, the intensity of 0.1 mA has better effects in activating PVN oxytocinergic neurons (Fig. 2D, E). Quantitative analysis revealed that both the peak and AUC of calcium signals were rapidly and pronouncedly increased with 2 Hz EA stimulation, and the 0.1 mA had the best effect (Fig. 2I, J). Then, we chose 0.1 mA and different frequencies to stimulate at ST36. Notably, the GCaMP signals revealed that 2 Hz EA stimulation at left ST36 for 2 s induced a potent elevation of photometric Ca^2+^ signals, whereas 10 Hz EA did not have this effect (Fig. 2F, G). Quantitative analysis revealed that both the peak and AUC of calcium signals were rapidly and pronouncedly increased during 2 Hz EA, while 10 Hz EA stimulation failed to do so (Fig. 2K, L). We also tried other parameters to further verify the remarkable effects of lower frequency EA on PVN oxytocinergic neurons (Figure S2C-J).

To validate the obvious activation of PVN oxytocinergic neurons by 2 Hz EA, we performed 0.1 mA EA stimulation for 30 min at the ST36 acupoint located on the left hind limb of the mice. The PVN of mice was then stained for OXT and c-Fos. Consistent with the fiber photometry data, a markedly higher proportion of OXT-ir neurons co-expressed c-Fos following 2 Hz EA compared to 0 Hz EA stimulation, whereas 10 Hz EA failed to activate oxytocinergic neurons (Fig. 2H, M). These findings collectively demonstrate that lower frequency EA activates more oxytocinergic neurons in the PVN, suggesting that the frequency of EA largely determines the magnitude of PVN oxytocinergic neurons’ engagement.

### Electroacupuncture exerts an analgesic effect in inflammatory pain

**Fig. 3:**
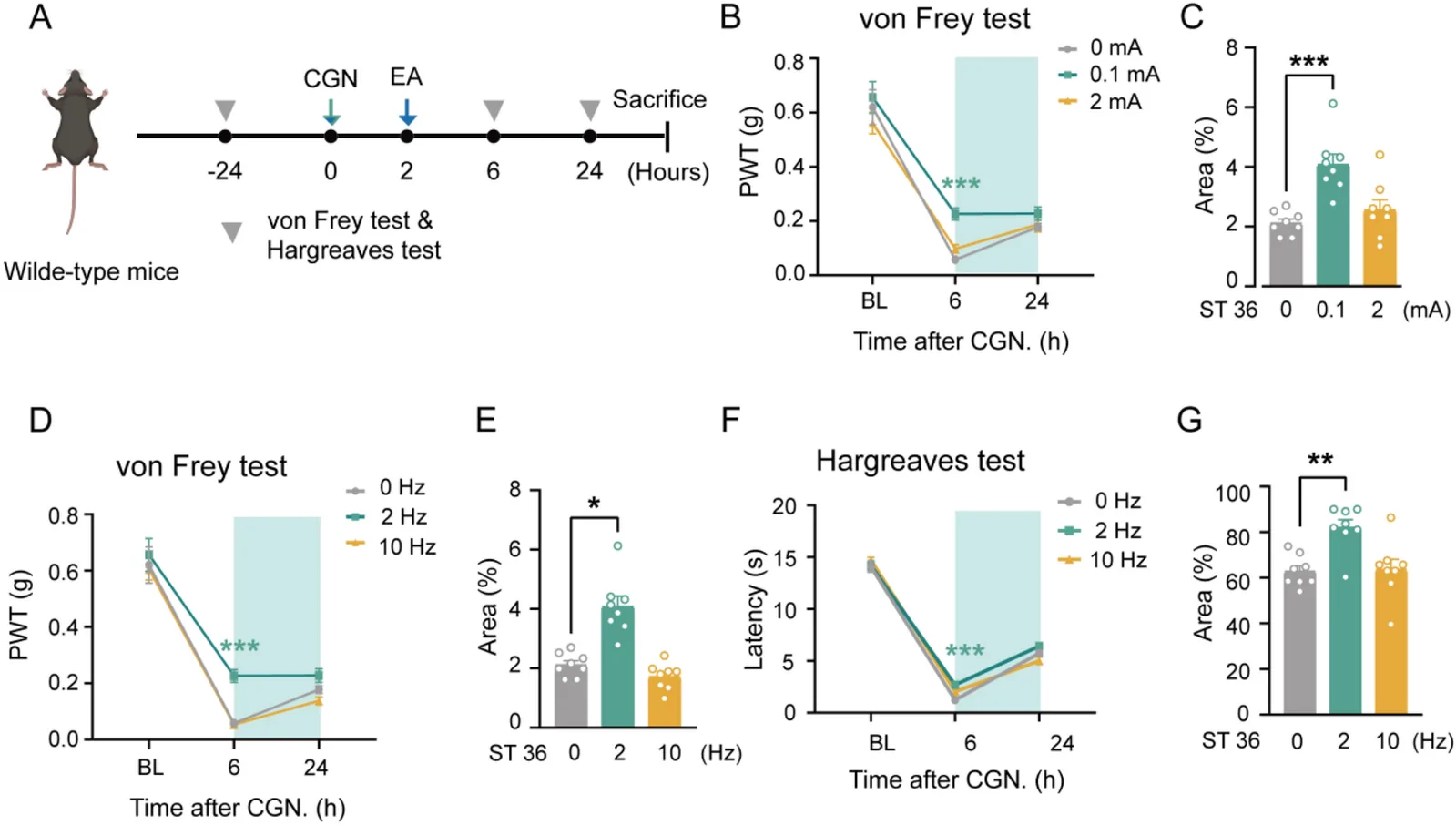
Electroacupuncture exerts an antinociceptive effect in inflammatory pain. **A** Schematic of the experimental procedure. **B** Mechanical pain threshold after EA stimulation of 2 Hz with different intensities in CGN mice, n = 8 mice per group. **C** Normalized AUC of PWT during 6 to 24 h from **B**, n = 8 mice per group. **D** Mechanical pain threshold after EA stimulation of 0.1 mA with different frequencies in CGN mice, n = 8 mice per group. **E** Normalized AUC of PWT during 6 to 24 h from **D**, n = 8 mice per group. **F** Thermal pain threshold after EA stimulation of 0.1 mA EA at different frequencies in CGN mice, n = 8 mice per group. **G** Normalized AUC of latency during 6 to 24 h from **F**, n = 8 mice per group

We have confirmed that 2 Hz 0.1 mA EA could dramatically promote the activation of PVN oxytocinergic neurons. To elucidate whether the stimulation exerts effects on modulating inflammatory pain, we next evaluated the analgesic efficacy of EA in a CGN-induced inflammatory pain model. EA was administered for 30 min beginning 2 h after CGN injection, and nociceptive thresholds were assessed at 6 h post-injection and again on the following day (Fig. 3A).

The results showed that 2 Hz 0.1 mA EA stimulation exerted a clear analgesic effect on inflammatory pain, while the 2 Hz with 0 mA or 2 mA EA failed to elicit any measurable analgesic response (Fig. 3B, C). To determine the frequency dependence of this effect, we perform 0.1 mA with various frequencies of EA stimulation. Notably, only the 2 Hz stimulation, rather than 10 Hz, significantly alleviated CGN-induced mechanical allodynia (Fig. 3D, E). Additional parameters testing at the ST36 acupoint revealed that the 2 Hz 0.5 mA EA stimulation, rather than the 100 Hz 0.1 mA, was effective in reducing pain behaviors (Figure S3A-D). These findings collectively indicate that lower frequency EA optimally mitigates inflammatory pain hypersensitivity.

To verify the analgesic effect of EA, we further examined thermal nociception. Consistent with the mechanical results, 2 Hz 0.1 mA EA also had a significant alleviating effect on CGN-induced thermal hyperalgesia (Fig. 3F, G). Given that the ipsilateral limb was used for both EA intervention and nociceptive testing, we further examined the pain threshold of the ipsilateral hind paw in naïve mice 4 h after EA treatment. Notably, EA application had no effect on the baseline pain threshold in the ipsilateral side of naïve mice (Figure S3E-F). In parallel with the results of chemogenetic activation of PVN oxytocinergic neurons, EA has a slight effect on CGN-induced paw edema (Figure S3G-H). Collectively, these results demonstrate that lower intensity and lower frequency EA elicits potent analgesic effects in this inflammatory pain model, in concordance with the parameters that maximally activate PVN oxytocinergic neurons. The EA parameters of 2 Hz 0.1 mA were selected for the following research.

### Electroacupuncture-induced analgesia is mediated by PVN oxytocinergic neurons

**Fig. 4:**
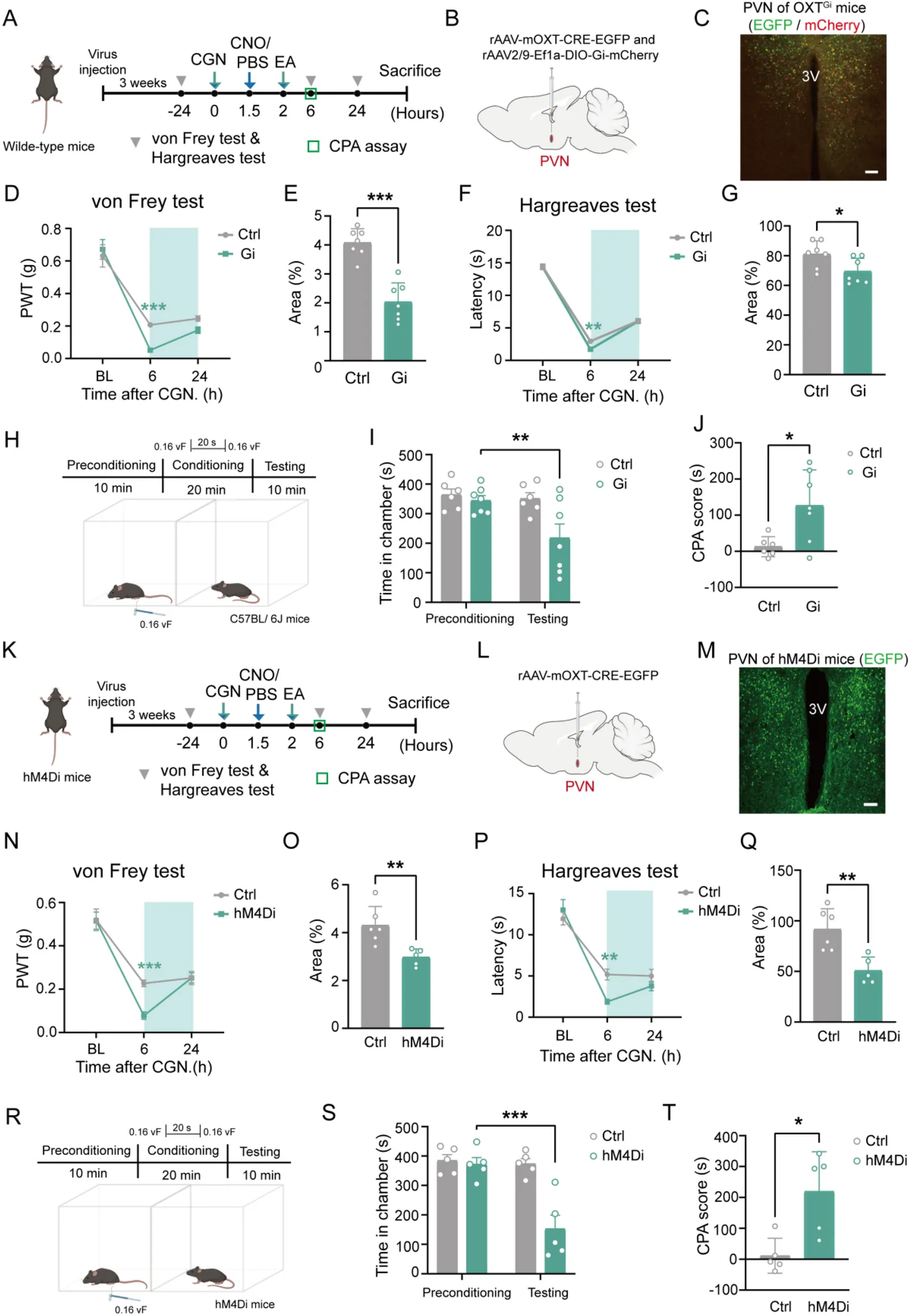
Chemogenetic silence of the PVN oxytocinergic neurons weakens the analgesic effect of electroacupuncture. **A** Schematic of the experimental procedure. **B** Schematic of microinjection of rAAV-mOXT-CRE-EGFP and rAAV2/9-Ef1a-DIO-Gi-mCherry into the PVN region of WT mice. **C** Representative images validate EGFP and mCherry expression in PVN (scale bar = 100 μm). **D** Mechanical pain threshold after 2 Hz 0.1 mA EA stimulation of mice in the Gi or the Ctrl group, n = 7 mice per group. **E** Normalized AUC of PWT during 6 to 24 h from (D), n = 7 mice per group. **F** Thermal pain threshold after 2 Hz 0.1 mA EA stimulation of mice in the Gi group or the Ctrl group, n = 7 mice per group. **G** Normalized AUC of latency during 6 to 24 h from **F**, n = 7 mice per group. **H** Schematic of the CPA experimental procedure. **I** Time spent in the stimulation chamber of mice in the Gi group or the Ctrl group in the CPA experiment, n = 6–7 mice per group. **J** CPA scores of mice in the Gi group or the Ctrl group in the CPA experiment, n = 6–7 mice per group. **K** Schematic of the experimental procedure. **L** Schematic of microinjection of rAAV-mOXT-CRE-EGFP into the PVN region of the *hM4Di* mice. **M** Representative images validate EGFP expression in PVN of the *hM4Di* mice (scale bar = 100 μm). **N** Mechanical pain threshold after 2 Hz 0.1 mA EA stimulation of mice in the hM4Di or the Ctrl group, n = 5–6 mice per group. **O** Normalized AUC of PWT during 6 to 24 h from **N**, n = 5–6 mice per group. **P** Thermal pain threshold after 2 Hz 0.1 mA EA stimulation of mice in the hM4Di group or the Ctrl group, n = 5–6 mice per group. **Q** Normalized AUC of latency during 6 to 24 h from **P**, n = 5–6 mice per group. **R** Schematic of the CPA experimental procedure. **S** Time spent in the stimulation chamber of mice in the hM4Di group or the Ctrl group in the CPA experiment, n = 5 mice per group. **T** CPA scores of mice in the hM4Di group or the Ctrl group in the CPA experiment, n = 5 mice per group

Given that lower frequency EA significantly enhanced the activity of PVN oxytocinergic neurons, we next investigated whether these neurons were functionally required for EA-induced analgesia (Fig. 4A). To achieve selective inhibition of PVN oxytocinergic neurons, mixed rAAV-mOXT-CRE-EGFP-WPRE-bGH and rAAV2/9-Ef1a-DIO-Gi-mCherry-WPRE (1:1) were injected into the bilateral PVN of mice (Fig. 4B). This approach enabled Cre-dependent expression of the inhibitory DREADD receptor Gi in oxytocinergic neurons. We verified EGFP and mCherry expression in the specific region of the PVN (Fig. 4C).

Following a 3-week recovery period, mice were subjected to CGN-induced inflammatory pain and subsequently stimulated with 2 Hz EA at the ST36 acupoint. CNO (1 mg/kg) or equal PBS was intraperitoneally administered 30 min prior to EA, respectively in mice of the Gi or the Ctrl group (Fig. 4A), ensuring that silencing of oxytocinergic neurons was achieved during the EA stimulation. Behavioral assays revealed that inhibition of PVN oxytocinergic neurons markedly attenuated the analgesic effect of 2 Hz EA, as evidenced by decreased paw withdrawal thresholds (Fig. 4D, E) and shortened heat withdrawal latencies (Fig. 4F, G) compared to the mice of the Ctrl group. These findings indicate that PVN oxytocinergic activity is essential for the analgesic efficacy of lower frequency EA.

Besides its sensory dimension, pain also has emotional and motivational components that are indispensable in pain assessment. Mechanical allodynia test and thermal hyperalgesia test reflected the pain threshold of mice, but were not sufficient to reflect the mice's emotional response to pain stimuli. To evaluate the aversive emotion for pain and the role of oxytocinergic neurons in it, we conducted a well-established two-chamber CPA assay at 6 h after CGN injection (Fig. 4H).

When PVN oxytocinergic neurons were chemogenetically silenced before EA stimulation, the mice exhibited a significant aversion to the stimulus-paired chamber (Fig. 4I), suggesting that the inhibition of these neurons diminished the EA-induced attenuation of pain-related aversive behavior. To quantify these results, CPA scores were calculated. Mice in the Gi group displayed significantly higher CPA scores compared with the mice in the Ctrl group (Fig. 4J). Collectively, these results indicate that the anti-nociceptive and anti-aversive effects of EA depend on the function of oxytocinergic neurons in the PVN.

To further corroborate these findings, we employed transgenic *hM4Di* mice (Fig. 4K). The bilateral PVN of these mice was injected with rAAV-mOXT-CRE-EGFP-WPRE-bGH virus, resulting in selective EGFP expression within the PVN region (Fig. 4L, M). Consistent with previous results, chemogenetic silence of the PVN oxytocinergic neurons markedly attenuated the analgesic effect of EA in mechanical and thermal pain (Fig. 4N–Q). Similarly, CPA testing revealed a pronounced pain aversion in *hM4Di* mice (Fig. 4R–T). Hence, these findings strongly suggest that PVN oxytocinergic neurons play a pivotal role in mediating the analgesic effects of EA.

### PVN oxytocinergic neurons projecting to ARC are activated during electroacupuncture

**Fig. 5:**
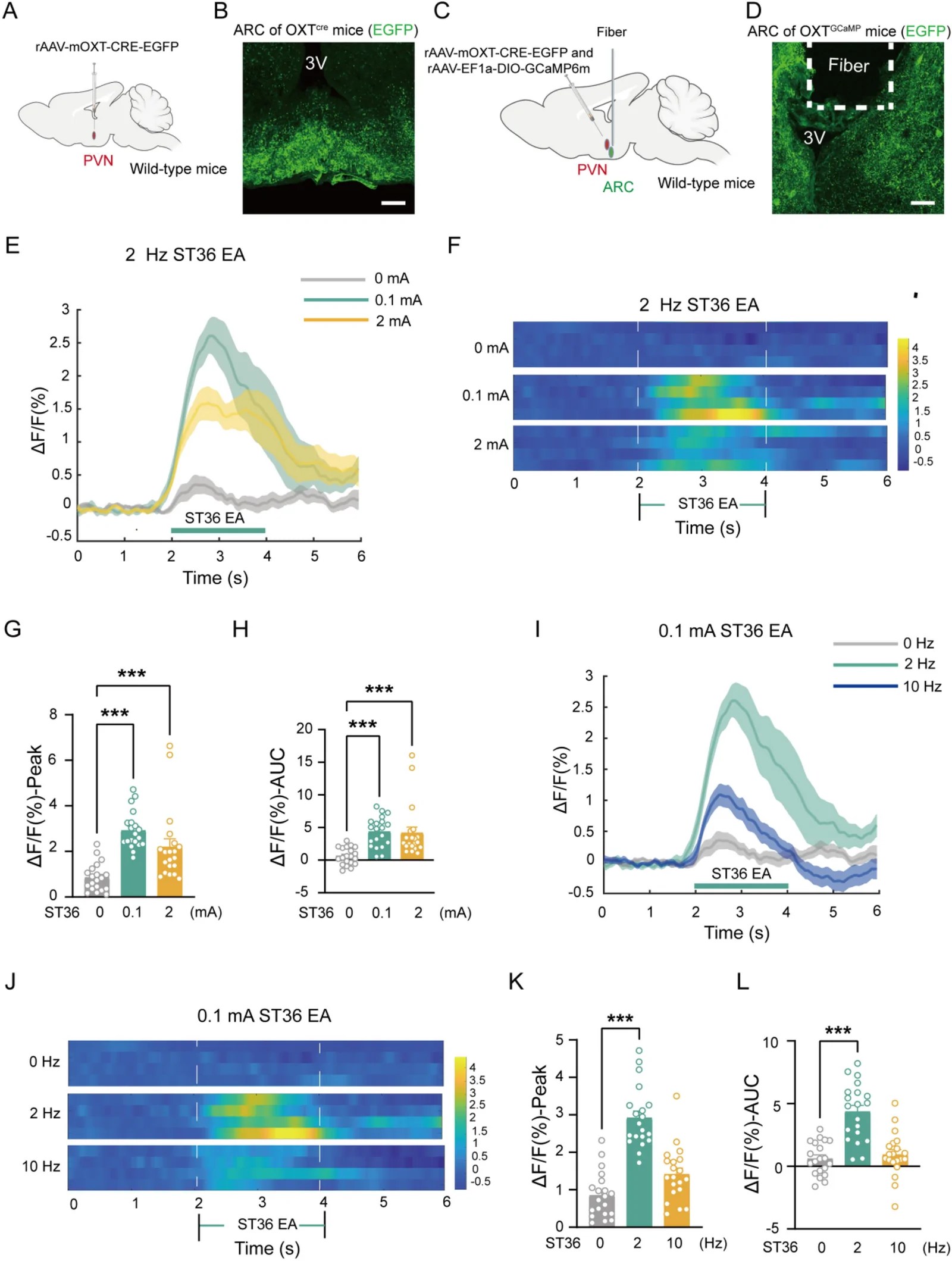
Electroacupuncture activated oxytocinergic neurons in the PVN projecting to the ARC. **A** Schematic of microinjection of rAAV-mOXT-CRE-EGFP into the PVN region of WT mice. **B** Representative images validate EGFP expression in the ARC of WT mice (scale bar = 50 μm). **C** Schematic of microinjection of rAAV-mOXT-CRE-EGFP and rAAV-EF1a-DIO-GCaMP6m into the PVN region and the implantation of a fiber into the ARC region in WT mice. **D** Representative images validate EGFP expression and fiber implantation in the ARC of WT mice (scale bar = 50 μm). **E** Typical examples of traces and **F** heat maps of Ca^2+^ transients in ARC induced by EA stimulus of 2 Hz for 2 s. **G** Quantitative summary of Ca^2+^ transients in peak amplitude and **H** AUC before and after EA stimulation of 2 Hz with different intensities, n = 20 trials from 4 mice per group. **I** Typical examples of traces and **J** heat maps of Ca^2+^ transients in ARC induced by EA stimulus of 0.1 mA for 2 s. **K** Quantitative summary of Ca^2+^ transients in peak amplitude and **L** AUC before and after EA stimulation of 0.1 mA with different frequencies

We next explored the distal target of PVN oxytocinergic neurons in EA-induced analgesia. Among the downstream of the PVN oxytocinergic neuron projections, the ARC drew our attention. The ARC has previously been shown to be a critical mediator in the frequency-dependent analgesic effects of EA. Studies reported that lesion of the ARC abolishes the pain relief induced by low-frequency EA but not high-frequency EA [10, 39]. We injected rAAV-mOXT-CRE-EGFP-WPRE-bGH into the bilateral PVN, and robust EGFP-positive axonal projections were observed in the ARC (Fig. 5A, B).

To further verify whether the PVN-ARC oxytocinergic neurons are involved in the frequency-specific EA effects, we conducted in vivo real-time Ca^2^⁺ fiber photometry recordings of oxytocinergic axonal terminals in the ARC during EA stimulation. Mixed rAAV-mOXT-CRE-EGFP-WPRE-bGH and rAAV-EF1a-DIO-GCaMP6m-WPRE-hGH were stereotaxically injected into the PVN of mice, followed by implantation of an optical fiber targeting the ARC (Fig. 5C, D). The changes in GCaMP fluorescence in response to EA stimulation were recorded. The results demonstrated that EA stimulation at 2 Hz significantly increased GCaMP fluorescence intensity, characterized by elevated peak amplitudes and larger AUC (Fig. 5E–H). This activation was most pronounced at an intensity of 0.1 mA, confirming that lower intensity, lower frequency EA optimally engages PVN-ARC oxytocinergic pathways.

Furthermore, when the frequency of EA was varied at an intensity of 0.1 mA, only 2 Hz stimulation effectively activated PVN-ARC oxytocinergic terminals, whereas 10 Hz stimulation produced no significant change (Fig. 5I–L). Consistent results were observed with other parameter combinations—specifically, 2 Hz at 0.5 mA evoked robust calcium responses, whereas 100 Hz at 0.1 mA did not (Figure S4A-H). Altogether, these findings indicate that lower frequency EA selectively activates the PVN-ARC oxytocinergic neurons, which may serve as a key neural substrate underlying the frequency-specific analgesic effects of EA.

### Electroacupuncture-induced analgesia depends on the OXTR system in the ARC

**Fig. 6:**
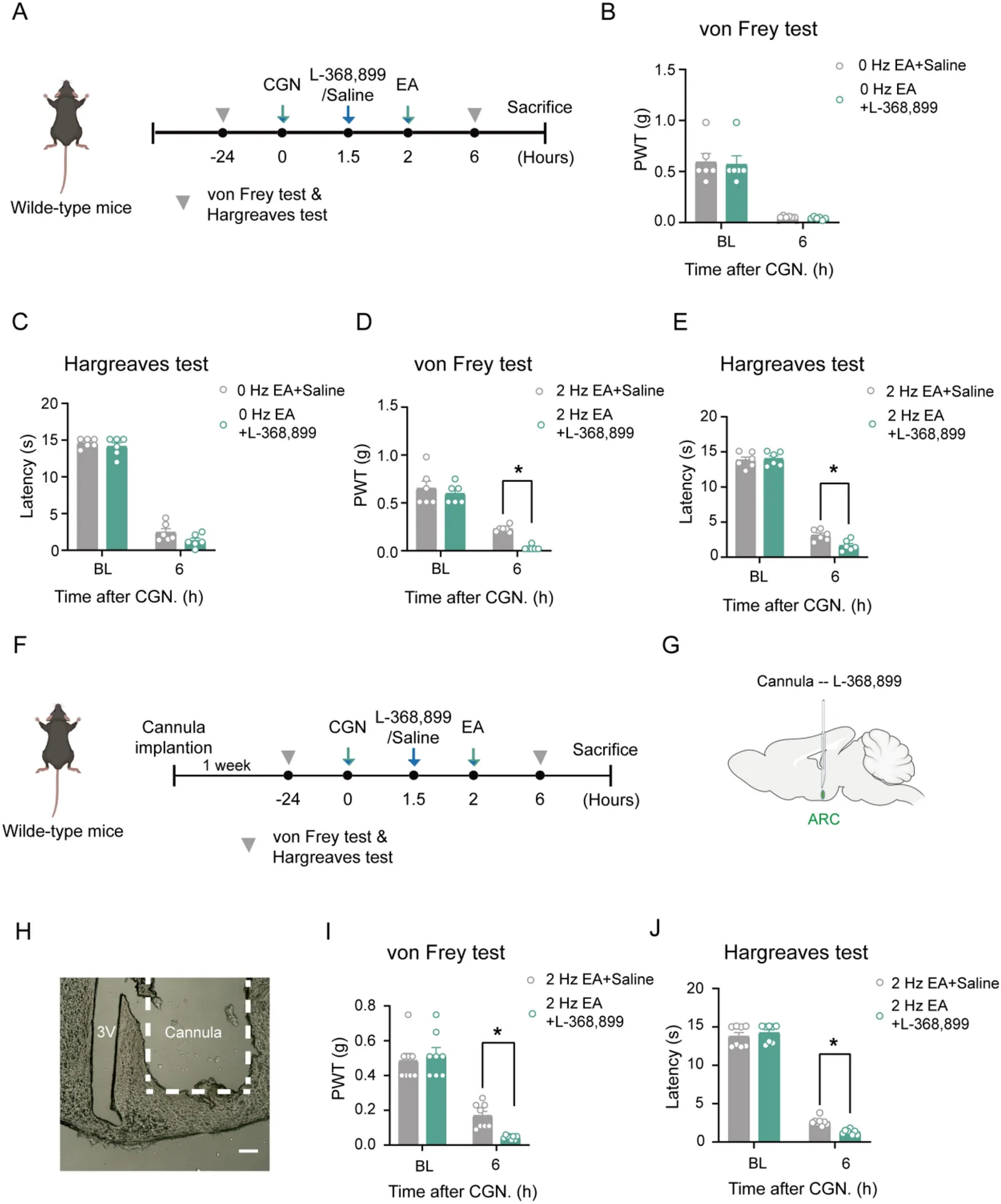
Electroacupuncture-induced analgesia depends on the OXTR system in the ARC. **A** Schematic of the experimental procedure for systemic administration of L-368,899. **B** Mechanical pain threshold and **C** Thermal pain threshold after systemic administration of L-368,899 or saline and 0 Hz 0 mA EA stimulation in the left paw of mice, n = 6 mice per group. **D** Mechanical pain threshold and **E** Thermal pain threshold after systemic administration of L-368,899 or saline and 2 Hz 0.1 mA EA stimulation in the left paw of mice, n = 6 mice per group. **F** Schematic of the experimental procedure for local administration of L-368,899 into the ARC region. **G** Schematic of the implantation of the cannula and administration of L-368,899 into the ARC of mice. **H** Representative images validate the location of the cannula into the ARC of mice (scale bar = 100 μm). **I** Mechanical pain threshold and **J** Thermal pain threshold after local administration of L-368,899 or saline into the ARC region and 2 Hz 0.1 mA EA stimulation in the left paw of mice, n = 8 mice per group

To exert oxytocinergic neuronal effects, released OXT binds to local OXTR, which are expressed within or in very close distance to the target region [40]. To investigate whether the pain relief effect of EA is mediated through the OXTR, we first systematically applied L-368,899 (5 mg/kg), a selective, non-peptide OXTR antagonist [41], 30 min prior to EA stimulation. The mechanical and thermal pain thresholds of mice were measured at 6 h to determine whether blockade of OXTR influenced EA’s effects (Fig. 6A). The results showed that the L-368,899 had no discernible effect on pain behaviors in animals receiving 0 Hz EA (Fig. 6B, C). Remarkably, systematic administration of L-368,899 completely abolished the 2 Hz EA-induced analgesia for both mechanical (Fig. 6D) and thermal (Fig. 6E) pain. These results indicated that the analgesic effects of EA depend on the endogenous OXTR system.

To further investigate the role of the ARC OXTR system in EA analgesia, we locally injected the OXTR antagonist L-368,899 into the ARC region 30 min before EA stimulation. The mechanical and thermal pain thresholds of mice were measured at 6 h after the establishment of the inflammatory pain model (Fig. 6F–H). The results showed that specifically blocking the target of oxytocinergic neurons in the ARC did not affect the mechanical and thermal pain thresholds of mice receiving 0 Hz EA (Figure S4I-J). However, in mice receiving 2 Hz EA stimulation, local blockade of OXTRs in the ARC region significantly reduced the analgesic effect of EA on mechanical and thermal pain (Fig. 6I, J). These results indicate that the OXTRs in the ARC are essential for the frequency-specific EA analgesia.

## Discussion

Our study elucidates that the PVN oxytocinergic neurons serve as critical mediators of the frequency-specific analgesic effects of EA stimulation, with a potent regulatory role in inflammatory pain. Notably, the PVN oxytocinergic neurons were activated by lower-frequency EA stimulation. Activating PVN oxytocinergic neurons has an effect similar to that of EA on pain. Furthermore, we discovered that PVN-ARC oxytocinergic neurons were activated during lower-frequency EA stimulation. Blocking the function of OXTRs in the ARC reduced the analgesic effect of EA. These findings provide novel insights into the neural mechanisms underlying EA-induced, frequency-specific analgesia and highlight potential guidelines for optimizing EA parameters for pain relief.

CGNs are a family of sulfated polysaccharides extracted primarily from red seaweeds and are widely utilized in the food, pharmaceutical, and cosmetic industries due to their gelling, thickening, and stabilizing properties [42]. There are three main types of CGN: lambda, kappa, and iota. The lambda form does not gel strongly at room temperature and is injectable to induce an inflammatory response [43]. The injection of CGN into the sub-plantar region of the animals’ hind paw induces paw edema rapidly. A study reported that after 120 min of modeling, the right hind paws of rats showed varying degrees of edema, and histopathologic symptoms such as interstitial edema, inflammatory exudation, and vascular injury were observed in the model group [44]. CGN-evoked mechanical allodynia and thermal hyperalgesia also developed within 2 h [45]. Corresponding with previous investigations, CGN administration elicited persistent inflammatory pain behaviors and paw edema in mice (Figure S1A-F). The effects and mechanisms of oxytocinergic neurons in the PVN and EA analgesia were investigated following the model paradigm. Our observations align with previous evidence that OXT is believed to have a protective effect under pain conditions [46]. PVN oxytocinergic neurons are activated after the administration of CGN. Furthermore, activation of PVN oxytocinergic neurons attenuates both mechanical allodynia and thermal hyperalgesia in the CGN-induced inflammatory pain model.

It is well known that acupuncture exerts profound therapeutic benefits on various pain diseases in the clinical setting [47], and OXT is considered an important mediator in the analgesic process of EA. For example, injection of OXT into the ventricles of rats can significantly enhance the analgesic effect of EA, while injection of OXT antibodies into the ventricles to neutralize endogenous OXT significantly weakens the analgesic effect of EA [48]. In addition, EA stimulation can significantly increase the concentration of OXT in several brain regions related to central analgesia, including the periaqueductal gray, raphe magnus nucleus, caudate nucleus, thoracic and lumbar spinal cord, but did not alter OXT concentration in the anterior pituitary, posterior pituitary, and plasma [48]. The above research results indicate that OXT in the central nervous system plays an important role in the analgesic mechanism of EA [49]. The oxytocinergic neurons connecting to the central nervous system regions were mainly localized within the PVN [50]. Therefore, we speculate that EA may exert its significant analgesic effect by activating the oxytocinergic neurons in the PVN.

To verify this hypothesis, this study used in vivo calcium photometry and immunofluorescence staining and demonstrated that PVN oxytocinergic neurons are activated by lower-frequency EA stimulation, while higher-frequency EA failed to do so. Compared with manual acupuncture, EA provides more reliable and quantifiable stimulation, allowing precise control over parameters such as frequency, intensity, and duration. EA with different parameters can achieve different therapeutic effects via different mechanisms [10]. Prior findings indicated that the analgesic outcome of EA is strongly dependent on stimulation frequency [6]. We found that lower-frequency EA produced a robust analgesic effect in the CGN-induced inflammatory pain mouse model, rather than the higher-frequency EA stimulation. Moreover, chemogenetic inhibition of the PVN oxytocinergic neurons markedly reduced EA-induced analgesia.

As above, more oxytocinergic neurons in the PVN were activated at lower frequency and lower-intensity EA stimulation, which has better analgesic effects. These findings suggest that OXT release may serve as an indicator of EA efficacy under different stimulation parameters. Thus, quantification of OXT levels could potentially guide the optimal EA parameters for individualized pain management. With advancements in OXT detection technologies, such as the measurement of OXT in human saliva samples [51], it may become feasible to use OXT as a non-invasive biomarker of EA-induced analgesia in clinical practice. These insights support the development of precise, effective, and personalized EA treatment for pain modulation.

PVN neurons project extensively to multiple central nervous system regions involved in diverse physiological processes. Among these, the ARC has caught our attention, which has been proven to be vital in the analgesic effects of low-frequency EA stimulation. The ARC is a principal component in the endogenous opioid peptide system, in which β-endorphin-containing neurons are densely located [52]. Lesion of the ARC abolished analgesia induced by lower frequency EA but not that of higher frequency EA [10], implying a frequency-dependent regulatory mechanism. However, the upstream regions modulating ARC activity during EA-induced analgesia remain unclear. Previous work has shown that oxytocinergic neurons in the PVN directly innervate pro-opiomelanocortin (POMC) neurons in the ARC, which express OXTR [20]. In line with this anatomical connection, our data confirm that lower-frequency EA activates the PVN-ARC oxytocinergic pathway, which may subsequently influence endorphinergic signaling to the periaqueductal gray [9]. Blocking the function of the OXTR system in the ARC significantly alleviates the pain relief effects of EA, which highlights the important role of PVN-ARC oxytocinergic neurons in EA analgesia.

Nevertheless, our study has several limitations. First, EA was administered only once, and repeated stimulations are needed to evaluate the long-term therapeutic outcomes. Besides, in the CGN-induced inflammatory pain mouse model, the EA stimulation was performed 2 h after the CGN injection, corresponding to the onset of pain hypersensitivity [44, 45]. Thus, our paradigm primarily reflects an acute inflammatory pain state rather than chronic pain conditions. Lastly, the present study utilized only the CGN-induced model, and the sample size is limited. Future studies should extend to other pain paradigms and use a larger sample size to determine whether similar oxytocinergic mechanisms are conserved.

## Conclusions

In conclusion, our research highlights the PVN oxytocinergic neurons as critical neural substrates underlying the frequency-specific analgesia of EA, and these findings guide the selection of EA parameters in clinical pain management.

## Supplementary Information

Supplementary material 1.

## Data Availability

No datasets were generated or analysed during the current study.

## Abbreviations

ANOVA: Analysis of variance
ARC: Arcuate nucleus
AUC: Area under the curve
CNO: Clozapine-N-oxide
CGN: Carrageenan
CPA: Conditioned place aversion
EA: Electroacupuncture
NSAIDs: Non-steroidal anti-inflammatory drugs
OXT: Oxytocin
OXT-ir: OXT-immunoreactive
OXTR: Oxytocin receptor
PVN: Paraventricular nucleus
PFA: Paraformaldehyde
PBS: Phosphate-buffered saline
POMC: Pro-opiomelanocortin
SEM: Standard error of the mean
SON: Supraoptic nucleus
ST36: Stomach meridian 36
WHO: World Health Organization
