# Supplementary material for: Oxytocinergic neurons in the paraventricular nucleus mediate the analgesic effect of electroacupuncture in inflammatory pain
Source: Chin Med. 2026 Nov 11;21:237. doi: 10.1186/s13020-026-01513-x (PMC13753097; doi:10.1186/s13020-026-01513-x)
Supplement: Supplementary file 1 [file 13020_2026_1513_MOESM1_ESM.docx]

Supplementary Figure 1.

(A) Mechanical pain threshold in the left paw of mice in the CGN or the Saline group, n = 5 mice per group. (B) The mechanical pain threshold at 6 h after CGN or saline administration in the left paw of mice in the CGN or the Saline group, n = 5 mice per group. (C) The thermal pain threshold in the left paw of mice in the CGN or the Saline group, n = 5 mice per group. (D) The thermal pain threshold at 6 h after CGN or saline administration in the left paw of mice in the CGN or the Saline group, n = 5 mice per group. (E) The paw volume in the left paw of mice in the CGN or the Saline group, n = 5 mice per group. (F) The paw volume at 6 h after CGN or saline administration in the left paw of mice in the CGN or the Saline group, n = 5 mice per group. (G) Representative immunofluorescent staining of OXT (green) and c-Fos (red) in the PVN of the mice after CGN or saline administration (scale bar = 25 μm). White arrows indicate co-localization. (H) Percentage of c-Fos-positive OXT neurons after CGN or saline administration, n = 6 mice per group.

Supplementary Figure 2.

(A) The paw volume after CGN administration in the left paw of mice in the Gq or the Ctrl group, n = 6 mice per group. (B) Normalized AUC of paw volume during 6 to 24h from (A), n = 6 mice per group. (C) Typical examples of traces and (D) heat maps of Ca^2+^ transients in PVN oxytocinergic neurons induced by EA stimulus of 2 Hz 0.5 mA for 2 s. (E) Quantitative summary of Ca^2+^ transients in peak amplitude, and (F) AUC before and after EA stimulation of 2 Hz 0.5 mA, n = 20 trials from 4 mice per group. (G) Typical examples of traces and (H) heat maps of Ca^2+^ transients in PVN oxytocinergic neurons induced by EA stimulus of 100 Hz, 0.1 mA for 2 s. (I) Quantitative summary of Ca^2+^ transients in peak amplitude, and (J) AUC before and after EA stimulation of 100 Hz 0.1 mA, n = 20 trials from 4 mice per group.

Supplementary Figure 3.

(A) Mechanical pain threshold after EA stimulation of 2 Hz 0.5 mA in CGN mice, n = 6 mice per group. (B) Normalized AUC of PWT during 6 to 24h from (A), n = 6 mice per group. (C) Mechanical pain threshold after EA stimulation of 100 Hz 0.1 mA in CGN mice, n = 6 mice per group. (D) Normalized AUC of PWT during 6 to 24h from (C), n = 6 mice per group. (E) Mechanical pain threshold after EA stimulation of 2 Hz 0.1 mA in naïve mice, n = 8 mice per group. (F) Thermal pain threshold after EA stimulation of 2 Hz 0.1 mA in naïve mice, n = 8 mice per group. (G) The paw volume after EA stimulation of 2 Hz in CGN mice, n = 6 mice per group. (H) The paw volume after EA stimulation of 0.1 mA in CGN mice, n = 6 mice per group.

Supplementary Figure 4.

(A) Typical examples of traces and (B) heat maps of Ca^2+^ transients in ARC induced by EA stimulus of 2 Hz 0.5 mA for 2 s. (C) Quantitative summary of Ca^2+^ transients in peak amplitude, and (D) AUC before and after EA stimulation of 2 Hz 0.5 mA, n = 20 trials from 4 mice per group. (E) Typical examples of traces and (F) heat maps of Ca^2+^ transients in ARC induced by EA stimulus of 100 Hz 0.1 mA for 2 s. (G) Quantitative summary of Ca^2+^ transients in peak amplitude, and (H) AUC before and after EA stimulation of 100 Hz 0.1 mA, n = 20 trials from 4 mice per group. (I) Mechanical pain threshold, and (J) Thermal pain threshold after local administration of L-368,899 or saline administration and 0 Hz 0 mA EA stimulation in the left paw of mice, n = 8 mice per group.
